# Single+1 Port Robotic Splenectomy Using the da Vinci SP System—Experience of a Single Centre

**DOI:** 10.1002/rcs.70160

**Published:** 2026-04-07

**Authors:** Yeongsoo Jo, Seog Ki Min

**Affiliations:** ^1^ Department of Surgery Ewha Womans University Seoul Hospital Ewha Womans University College of Medicine Seoul Korea

**Keywords:** case series, da Vinci SP, robotic splenectomy, single‐port

## Abstract

**Background:**

Laparoscopic splenectomy is the standard procedure for benign splenic disease but remains challenging in splenomegaly or complex hilar anatomy. The da Vinci SP system offers enhanced dexterity and visualisation via a single port.

**Methods:**

We retrospectively reviewed six female patients who underwent single+1 port robotic splenectomy with the da Vinci SP system between August 2024 and January 2025. A 12‐mm assistant port allowed suction, retraction, and stapler use.

**Results:**

Mean age was 43.3 years and BMI 24.9 kg/m^2^. Indications included splenic cysts, indeterminate lesions, suspected disseminated peritoneal leiomyomatosis, and haemolytic anaemia. Mean operation and console times were 182.5 and 106.7 min, respectively; mean blood loss was 116.7 mL. One patient had splenic vein bleeding; no conversions or ≥ Clavien–Dindo II complications occurred. All patients were discharged by day 4.

**Conclusions:**

Single+1 port robotic splenectomy is feasible and safe; larger studies are warranted.

## 1 Introduction

Laparoscopic splenectomy has become the standard approach for elective removal of the spleen, particularly in patients with benign haematologic or cystic conditions, because of the well‐established benefits of laparoscopy over open surgery in terms of postoperative pain, length of hospital stay, and cosmetic outcomes [1, 2, 3, 4, 5, 6, 7, 8, 9, 10, 11]. Conventional multiport laparoscopic splenectomy (CLS) is the most widely performed laparoscopic technique.

Surgical techniques have advanced over recent decades, and many surgeons have introduced different kinds of laparoscopic procedures such as hand‐assisted laparoscopic surgery, reduced‐port laparoscopic surgery, and single‐incision laparoscopic surgery (SILS) [12, 13, 14, 15]. The approach to reduce the number of ports has potential advantages, including improved cosmetic outcomes, reduced postoperative incisional pain, and fewer wound‐related complications. Consequently, single‐port laparoscopic splenectomy is preferred by some surgeons [15, 16, 17]. However, this technique is limited by restricted instrument articulation, in‐line interference, and suboptimal ergonomics when operating in anatomically complex regions, such as the splenic hilum [18]. These limitations are exacerbated in patients with splenomegaly, dense perisplenic adhesions, or especially patients with extensive visceral fat, which hinders visualisation and safe dissection.

To overcome these constraints, robotic splenectomy using multiport systems offers improved dexterity, tremor filtration, and three‐dimensional magnified visualisation, and has been adopted in select institutions [19]. However, multiport robotic approaches require multiple incisions, potentially diminishing the cosmetic advantage and increasing the risk of port‐site complications. In recent years, single‐incision and single+1 port robotic techniques have gained attention as strategies to reduce surgical invasiveness while maintaining the precision of robotic platforms in various surgical fields [18, 20, 21, 22, 23]. The da Vinci SP surgical system, specifically designed for single‐incision surgery, features a flexible 25‐mm cannula that accommodates a fully wristed camera and three articulating robotic instruments through a single access point. Its unique design provides enhanced instrument triangulation, superior ergonomic control, and an unobstructed view within confined spaces—features that are particularly advantageous in splenic surgery. Furthermore, when complemented by an auxiliary 12‐mm port (single+1 port approach), the system enables flexible use of assistant‐driven devices, such as advanced energy devices, suction, staplers, or retractors without compromising the minimally invasive nature of the procedure.

Despite the growing interest in SP‐assisted procedures in gynaecology, urology and general surgery, the application of the da Vinci SP system to splenectomy remains extremely limited in the literature [24]. To the best of our knowledge, there are no detailed case series describing the technical feasibility, surgical modifications, or early outcomes of single+1 port robotic splenectomy using the da Vinci SP system.

The aim of this study is to describe our initial experience of single+1 port robotic splenectomy using the da Vinci SP system in six patients. We present the surgical techniques, perioperative outcomes, and intraoperative challenges encountered to provide early insight into the applicability, benefits, and potential limitations of this novel approach in the management of benign splenic diseases.

## 2 Materials and Methods

### 2.1 Patient Selection

This study retrospectively analysed six consecutive patients who underwent single+1 port robotic splenectomy using the da Vinci SP surgical system (Intuitive Surgical, Sunnyvale, CA, USA) between August 2024 and January 2025 at our institution, Ewha Womans University Seoul Hospital (Seoul, Republic of Korea). Indications for splenectomy included a large symptomatic splenic cyst, multiple splenic lesions, disseminated peritoneal parasitic myoma, and haemolytic anaemia.

All procedures were performed by a single hepato–biliary–pancreatic surgeon who was highly experienced in performing laparoscopic and robotic surgery using the da Vinci Xi system, including complex procedures such as major hepatectomy or pancreaticoduodenectomy. The surgeon had already accumulated substantial experience using the da Vinci SP system for cholecystectomy, bile duct resection, Roux‐en‐Y choledochojejunostomy, and left lateral sectionectomy. This case series represents the surgeon's initial clinical experience of performing splenectomy using the da Vinci SP surgical system.

### 2.2 Surgical Technique

**FIGURE 1:**
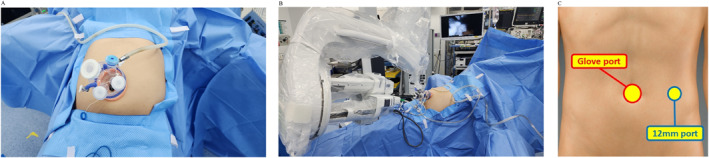
(A) Placement of the Glove Port at the umbilicus; (B) The patient is placed in the reverse Trendelenburg position, and the robotic docking is completed; (C) The 12‐mm assistant port at the left midclavicular line.

All procedures were performed using the patient in the supine position under general anaesthesia. A Glove Port (Nelis, Republic of Korea) was inserted through a transumbilical incision (2.5–3.0 cm) using the open method, and the SP trocar was introduced through this port to allow docking of the robotic system. An additional 12‐mm assistant port was placed at the same horizontal level as the umbilicus along the left midclavicular line. This port was used by the assistant to introduce energy devices, suction, and a stapler, as needed (Figure 1).

Following docking, the patient was placed in the reverse Trendelenburg position with a right‐side tilt to enhance exposure of the left upper quadrant. The procedure was carried out in the following sequence: (1) docking and patient positioning; (2) division of the gastrocolic ligament on the left side, and ligation/transection of the accessory splenic vessels and left gastroepiploic vessels; (3) ligation/transection of the short gastric vessels, followed by division of the gastrosplenic ligament; (4) dissection along the inferior border of the pancreas and division of the splenocolic ligament, followed by division of the branches of the splenic vessels; (5) isolation of the splenic hilum and division using a laparoscopic linear stapler; (6) division of the splenorenal and splenophrenic ligaments; and (7) final haemostasis and specimen retrieval.

**FIGURE 2:**
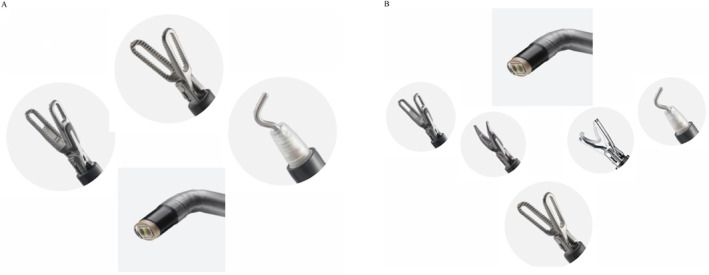
Arm configuration for below mode (A) and above mode (B).

**FIGURE 3:**
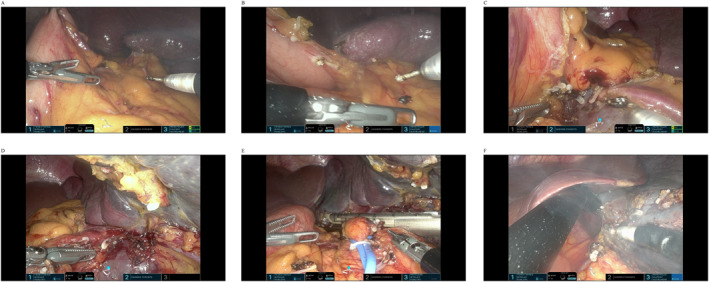
Intraoperative views: (A) Retraction of anterior stomach wall for exposure of gastrocolic ligament; (B) Retraction of posterior stomach wall; (C) Division of short gastric vessels; (D) Retraction of pancreas/transverse colon; (E) Division of splenic hilum with laparoscopic stapler; (F) Retraction of stomach/liver for hilar exposure.

For steps (1) and (2), the procedure was performed in the below mode using a monopolar hook in the 3 o'clock arm, fenestrated bipolar forceps in the 9 o'clock arm, and Cadiere forceps in the 12 o'clock arm (Figure 2A). Initially, the anterior wall of the stomach was elevated towards the ventral side of the patient using the Cadiere forceps to expose the gastrocolic ligament, facilitating step (1) (Figure 3A). Upon completion of this step, the posterior wall of the stomach was similarly grasped and elevated using the same instrument to allow access for step (2) (Figure 3B).

Beginning with step (3), the approach was converted to the above mode, and the Cadiere forceps were repositioned to the 6 o'clock arm. The 3 and 9o'clock arms were equipped with a monopolar hook, fenestrated bipolar forceps, Maryland bipolar forceps, or a polymer clip applier depending on the surgical need at each stage (Figure 2B). At this stage, the stomach was retracted using the 9 o'clock fenestrated bipolar forceps, and the short gastric vessels were divided followed by division of the gastrosplenic ligament (Figure 3C). For step (4), the pancreas or transverse colon was retracted using the 6 o'clock Cadiere forceps, or the spleen was elevated using the 9 o'clock fenestrated bipolar forceps to secure the operative field (Figure 3D). During step (5), isolation of the splenic hilum was facilitated, when necessary, by looping a rubber vessel loop around the hilum and retracting it towards the patient's lower right quadrant using the 6 o'clock Cadiere forceps to gently mobilise the pancreas by holding the loop. The hilum was meticulously divided by the assistant through the 12‐mm port using a laparoscopic linear stapler (Signia, Medtronic; 60‐mm tan cartridge) under direct robotic visualisation, with the stapler aligned as parallel to the splenic hilum as possible (Figure 3E). For steps (5) and (6), adequate visualisation was achieved by retracting the stomach or liver using the 9 o'clock arm (Figure 3F). If dense visceral fat or anatomical variations limited visibility, the assistant used the 12‐mm additional port to insert a laparoscopic instrument to aid visualisation of the surgical field. In all cases, we placed a Jackson‒Pratt drain catheter in the left subphrenic area through the additional port hole, and the specimen was placed in a laparoscopic retrieval bag and extracted through the umbilical incision, which was minimally extended as needed to facilitate extraction.

### 2.3 Data Collection

Demographic and clinical variables, including age, sex, body mass index, comorbidities, and prior surgical history, were reviewed. Surgical outcomes, such as operative time, estimated blood loss (EBL), length of hospital stay, intra‐ or postoperative complications (graded according to the Clavien‒Dindo classification), conversion to multiport laparoscopy or open surgery, and final pathological diagnosis, were also collected and analysed.

### 2.4 Ethics Approval

The study was approved by the Institutional Review Board of our institution (approval no. 2025‐07‐078), and the requirement for informed consent was waived due to the retrospective nature of the study.

## 3 Results

### 3.1 Case Presentation

Between August 2024 and January 2025, six female patients underwent single+1 port robotic splenectomy using the da Vinci SP system (Table 1). The mean age was 43.33 ± 14.75 years (range, 26–67 years) and the mean body mass index was 24.85 ± 4.41 kg/m^2^ (range, 17.41–31.02 kg/m^2^). Surgical indications were heterogeneous, and included symptomatic splenic cysts, indeterminate multiple splenic nodules, suspected disseminated peritoneal leiomyomatosis, and haemolytic anaemia.

**TABLE 1:** Single+1 robotic splenectomy case summary.

Case	Sex	Age	BMI	Chief complaint	Past history	Preoperative findings	Operation time (min)	Console time (min)	EBL (mL)	Discharge day (POD#)	Pathological diagnosis and spleen size
1	Female	26	17.41	Dyspepsia	None	Cystic mass (8 cm)	125	80	10	4	Epithelial cyst lined by squamous epithelium; 15.1 cm
2	Female	67	26.73	Abdominal pain	None	Multiple hypovascular lesions (up to 1.3 cm)	145	80	50	4	Multifocal haemangioma (haemangiomatosis); 12.5 cm
3	Female	50	24.72	OBGY consultation	None	Multiple myomas, suspected peritoneal parasitic myoma	265	80	20	3	Peritoneal leiomyoma; 11.0 cm
4	Female	30	31.02	Splenic hilum tumour	Appendectomy, C‐sec	4 cm ill‐defined lesion, r/o hamartoma	230	170	600	4	Splenic hamartoma (splenadenoma/splenoma); 6.4 cm
5	Female	45	24.97	Multiple splenic tumours	HTN, DL, RA, C‐sec	Multiple nodules (1.8 cm)	180	130	10	4	Haemangiomas, mild congestion; 17.1 cm
6	Female	42	24.24	Jaundice	Cholecystectomy, haemolytic anaemia	Splenomegaly	150	100	10	3	Congestion (clinical haemolytic anaemia); 19.0 cm

Abbreviations: BMI, body mass index; C‐sec, cesarean section; DL, dyslipidemia; EBL, estimated blood loss; HTN, hypertension; OBGY, obstetrics and gynaecology; POD, post‐operative day; RA, rheumatoid arthritis.

The mean total operative time was 182.50 ± 54.47 min (range, 125–265 min) and the mean console time was 106.67 ± 36.70 min (range, 80–170 min). The operation was longest in Case 3 (265 min), in which multidisciplinary cooperation among gynaecology and colorectal teams was required. In Case 4, intraoperative injury to the splenic vein during stapler application led to significant bleeding (EBL, 600 mL), prolonging the procedure. Splenomegaly (Massive splenomegaly was defined as a craniocaudal splenic length > 17 cm or spleen weight > 600 g, and supramassive splenomegaly as a length > 22 cm or weight > 1600 g [25].) in Case 5 contributed to the extended handling time, whereas Case 6, which also involved splenomegaly, was completed more efficiently, likely reflecting improved procedural proficiency.

The mean EBL was 116.67 ± 237.29 mL (range, 10–600 mL), with minimal blood loss in five of the six cases. All patients were discharged between postoperative days 3 and 4 with a mean hospital stay of 3.67 ± 0.52 days. No intraoperative conversions or postoperative complications were observed. According to the Clavien‒Dindo classification, none of the events were classified as Grade II or higher, including the case of postoperative pancreatic fistula and no blood transfusion was necessary during hospital stays. The final pathological analysis confirmed benign aetiologies in all cases, including epithelial cysts, haemangiomatosis, peritoneal leiomyomas, splenic hamartomas, and congestion secondary to haemolytic anaemia.

Postoperative follow‐up consisted of one outpatient visit at 1 month after discharge and one visit at 6 months thereafter; no further follow‐up was available. During the follow‐up period, there were no readmissions, emergency department visits, Clavien–Dindo complications, postoperative pancreatic fistula, or port‐site or umbilical hernia. All patients received appropriate preoperative or postoperative vaccinations in accordance with the institutional protocol. Vaccinations were coordinated with the infectious disease service; whenever feasible, they were administered approximately 2 weeks preoperatively, and when preoperative vaccination was not possible due to scheduling constraints, they were given about 2 weeks after discharge once the patient was clinically stable.

## 4 Discussion

Robotic splenectomy has evolved as a viable alternative to conventional laparoscopic or open approaches, offering enhanced visualisation, instrument articulation, and precision. CLS, though widely adopted, is often constrained by limited manoeuverability. In SILS, these limitations are further exacerbated by splenomegaly, perisplenic adhesions, or anatomically challenging regions such as the splenic hilum. Although multiport robotic systems have addressed some of these challenges, they still require multiple incisions, which may increase postoperative discomfort and the risk of port‐site complications. In contrast, the da Vinci SP system introduces a next‐generation platform for single‐incision surgery, minimising access trauma while retaining the ergonomic and visual advantages of robotic instrumentation.

In the present series of six cases of single+1 port robotic splenectomy, the SP system demonstrated excellent feasibility and safety across a range of benign splenic pathologies. The system's ability to deliver multi‐jointed, wristed instruments through a single transumbilical port facilitated fine dissection and vessel control in deep, narrow spaces, such as the splenic hilum. Superior visualisation was maintained throughout the procedures with stable camera control and unobstructed operative fields. Compared with conventional laparoscopic techniques, in which in‐line instrument crowding and suboptimal triangulation are frequently problematic, the SP platform allowed improved surgical dexterity and spatial freedom, even in patients with moderate splenomegaly.

A notable feature of our technique was the integration of an auxiliary 12‐mm port (i.e., single+1), which was selectively used by the assistant to introduce suction or energy devices, staplers, and additional retractors. This modification was particularly beneficial in enhancing intraoperative exposure and maintaining surgical efficiency, especially when dense visceral fat or vascular complexity demanded dynamic assistance. The single+1 port approach thus preserved the benefits of single‐incision surgery while addressing its limitations in exposure and instrumentation range. Importantly, none of the cases in this series required conversion to multiport or open surgery, further supporting the procedural stability and technical adequacy of the method.

Despite the overall favourable outcomes, we encountered some technical challenges. In Case 4, manipulation of the stapler led to an iatrogenic splenic vein injury, resulting in significant haemorrhage (EBL, 600 mL). This highlights the critical need for precise alignment and controlled force transmission when using stapling devices within the confined workspace of the da Vinci SP system. Nevertheless, haemostasis was successfully achieved robotically, and the procedure was completed without conversion. No patients in this case series experienced postoperative complications classified as Clavien‒Dindo grade II or higher, underscoring the overall safety of the approach.

As with all advanced surgical platforms, there is an adaptation period associated with gaining familiarity with the da Vinci SP system. In our small series, we noted an apparent reduction in operative complexity over time; for instance, Case 6, despite splenomegaly comparable to Case 5, was completed with a shorter console time and fewer technical challenges. However, given the very limited sample size and heterogeneity of the cases, these observations should be interpreted cautiously and cannot be taken as evidence of a learning curve. Formal assessment would require a larger consecutive cohort and appropriate analyses (e.g., trend testing or change‐point modelling) to account for case mix and procedural variability. Nonetheless, our descriptive experience is consistent with prior reports suggesting that proficiency in reduced‐port robotic techniques may be achieved after a brief initial adjustment period [26, 27].

To further contextualise the da Vinci SP system, it is useful to contrast it with other reduced‐port and conventional multi‐port robotic systems. Conceptually, the SP system enables camera and instrument deployment through a single access point with internal triangulation via SP‐specific cannula and arm articulation, whereas the da Vinci Single‐Site approach (implemented on a multi‐port platform) relies on a dedicated single‐site instrument set and may offer less instrument articulation and versatility for certain manoeuvres. In a comparative case–control study in early‐stage endometrial cancer, single‐incision robotic hysterectomy using the da Vinci SP system demonstrated perioperative outcomes broadly comparable to single‐incision robotic hysterectomy using the da Vinci Single‐Site approach without major differences in surgical outcomes, supporting the feasibility of an SP‐based reduced‐incision approach when appropriately selected [28]. Likewise, although derived from a different specialty, a meta‐analysis comparing multi‐port Xi with the da Vinci SP system or the da Vinci Single‐Site approach for radical prostatectomy reported generally similar operative time, blood loss, and postoperative complication rates across platforms, while noting that some outcomes may differ depending on platform and context [29]. Taken together, the available comparative literature suggests that the choice among the da Vinci SP system, the da Vinci Single‐Site approach, and the da Vinci Xi systems should be individualised based on procedural requirements, surgeon's experience, and patient‐specific factors; for splenectomy, the da Vinci SP system may offer a reduced‐incision configuration with SP‐specific ergonomic advantages, but careful case selection and technical standardisation remain important.

This study has several limitations and should be interpreted as a preliminary technical case series rather than an outcome study. First, the retrospective design and small number of cases preclude broad generalisation. Second, all procedures were performed by a single surgeon at a medium‐volume centre, potentially limiting reproducibility in lower‐volume or less experienced settings. Third, the study focused exclusively on benign disease, and the utility of this approach in malignant or trauma‐related splenic pathology remains to be investigated. Finally, the lack of a comparative control group (e.g., CLS, SILS, or multiport robotic splenectomy) prevents direct evaluation of the relative efficacy, cost‐effectiveness, and patient‐centred outcomes, such as postoperative pain and cosmesis.

In conclusion, single+1 port robotic splenectomy using the da Vinci SP system is a feasible and safe approach for selected patients, especially in patients with normal to mildly enlarged spleens. Although an adult case report has demonstrated the feasibility of robotic single‐port plus one assist port splenectomy using the da Vinci SP system, evidence to date has largely been limited to isolated reports [30]. In this context, our consecutive institutional experience adds a case‐series level description of the SP+1 configuration, including standardised technical steps and short‐term perioperative outcomes, which may help inform future larger studies. Further studies with larger cohorts and prospective controlled designs are warranted to define the role of the da Vinci SP system in routine and complex splenectomy. Future research should also explore the long‐term outcomes, incisional hernia rates, and patient satisfaction compared with other minimally invasive modalities.

## Author Contributions

**Yeongsoo Jo:** methodology, formal analysis, investigation, writing – review and editing. **Seog Ki Min:** conceptualization, methodology, writing – review and editing, supervision.

## Funding

The authors have nothing to report.

## Consent

The authors have nothing to report.

## Conflicts of Interest

The authors declare no conflicts of interest.

## Permission to Reproduce Material From Other Sources

The authors have nothing to report.

## Data Availability

The datasets generated during the current study are available from the corresponding author on reasonable request.
